# Experimental Investigation on Floating Solar-Driven Membrane Distillation Desalination Modules

**DOI:** 10.3390/membranes11050304

**Published:** 2021-04-21

**Authors:** Qingxiu Miao, Yaoling Zhang, Shuo Cong, Fei Guo

**Affiliations:** School of Energy and Power Engineering, Dalian University of Technology, Dalian 116024, China; miaoqxiu@126.com (Q.M.); emilyzhang2016@126.com (Y.Z.); congshuo@mail.dlut.edu.cn (S.C.)

**Keywords:** membrane distillation, solar-driven, configurations, floating

## Abstract

Membrane distillation (MD) processes need a relatively mild temperature gradient as the driving force for desalination. In the field, it is reasonable to utilize solar energy as the heat source for the feed, and seawater as the infinite cold source for condensation. Solar-driven MD provides a route for the practical application of seawater desalination at a small scale. In this work, we focus on floating MD modules with a solar heating bag as the power source, and perform proof-of-principle experiments on the MD performance under various conditioning parameters, including feed flow rate, feed temperature, salinity, air gap, and sea waves. The results indicate that floating solar-driven MD modules are feasible in terms of permeate flux and salt rejection ratio, and the upward evaporation MD configuration leads to a better performance in terms of permeate flux. The simulation and experiments also show that the natural sea waves disturb the heating bag and the MD module floating on the surface of seawater, and effectively enhance the feed circulation and transport in the system.

## 1. Introduction

Membrane distillation (MD) is a membrane separation process driven by the saturation vapor pressure drop across the hydrophobic membrane. It has been used for seawater desalination [1,2], industrial wastewater treatment [3], and removal of heavy metal ions from wastewater [4,5]. There are four major configurations of the MD system that have been proposed, which are direct contact membrane distillation (DCMD), air gap membrane distillation (AGMD), sweep gas membrane distillation (SGMD), and vacuum membrane distillation (VMD). In the AGMD system, a condensing surface on the permeate side is separated from the membrane surface by the air, which leads to higher thermal efficiency compared to DCMD. Besides, the structure of this system is less complex compared with SGMD and VMD systems.

Since the difference in vapor pressure varies exponentially with the temperatures of the feed and permeate in the MD process, according to Antoine’s equation [6], low-grade heat energy can be considered as its heat source, such as industrial waste heat [7,8]. Solar energy is one kind of low-grade and renewable clean energy. In recent years, using solar energy as the heat source of MD for seawater desalination has been studied by many researchers [9,10,11,12,13,14]. One way to use solar energy as the heat source of MD for seawater desalination is to heat the seawater in the heat pipe solar collector (Figure S1a) and then produce the drinkable water in the MD device [15,16]. It can be used as a small water supply station on an island. A major disadvantage of this method is that it is difficult to move the device.

In many rural areas with insufficient infrastructures, residents use solar heating bags (Figure S1b) instead of the heat pipe solar collector to heat water. The use of this heating technology is of great significance to improve the quality of life in developing countries. The solar heating bag is made of ethylene-vinyl chloride copolymer (EVC), which is soft, easy to carry, and suitable for functions in the field. Under sufficient sunlight, the temperature of the water can reach 60 °C. Depending on these advantages, we propose another way to use solar energy as the heat source of MD to produce drinkable water, which is using the solar heating bag to heat seawater. Compared to the heat pipe solar collector, using this method, the MD device is easy to move. Compared with the hydrophobic membrane [17,18,19] and the support net [20] with photothermal conversion properties, this way has a higher thermal energy utilization rate [21,22].

In addition, MD also needs a cold source to generate temperature gradiant and condense the water vapor into liquid phase. For air gap membrane distillation (AGMD) configuration, there is a condensing plate that makes it possible to directly use seawater as the coolant. As a rich marine resource, seawater has a relatively stable surface temperature in the same area [23,24], which could be considered as a cold source for MD. Besides, the temperature polarization near the hydrophobic membrane surface can decrease the permeate flux of the MD module. Wave energy is another low-grade and renewable energy [25,26]. Floating on the surface of the seawater, sea waves can provide a driving force for the internal circulation flow of the MD module, which can reduce the temperature polarization, and enhance the performance of the MD module in terms of permeate flux. Based on such resources and logic, we propose a novel MD configuration. In this configuration, solar energy is used as the heat source, and the seawater is used as the cold source. Additionally, wave energy as a driving force is used to enhance performance. It is energy-saving, foldable, and adaptable to the environment. This MD configuration can produce drinkable water only by natural resources.

In this work, we proposed a novel solar-driven MD system floating on the seawater surface. The system can generate drinkable water by natural resources which can be used in some field works. The proof of principle configurations were compared in terms of permeate flux and salt rejection ratio. Experimental tests and numerical simulations were applied to study the permeate flux and temperature distribution of the optimal configuration with the effect of sea waves. Finally, the performance of the optimal configuration under high salt solution was studied. The results verify that the novel solar-driven MD device can work effectively on the seawater surface.

## 2. Experimental

### 2.1. Membrane Characterization

Commercial hydrophobic polytetrafluoroethylene (PTFE) membranes were used in this work, which were purchased from Membrane Solution LLC. A scanning electron microscope (SEM, FEI, QUANTA450, Hillssboro, OR, USA) was used to characterize the surface morphology of the membranes. Before the test, the sample membrane was coated with a thin conductive layer of gold by a sputter coating apparatus (Q150T, Quorum Emitech & Polaron) for 60 s.

The membrane needs to withstand pressure fluctuations, so it must have suitable liquid entry pressure (LEP). In this work, the LEP of the PTFE membrane was tested by a custom-design unit, as described in detail elsewhere [6,27]. The thickness of the membrane was 170 ± 20 μm. Detailed information regarding the measurement of the membrane thickness can be found in previous studies [27,28].

The hydrophobicity of the PTFE membrane can be characterized by the static water contact angle (WCA). A goniometer (YIKE-360A, Chengde Yike Experimental Instrument Co., Ltd. Chengde, Hebei, China) was used to measure the WCA. The membrane was cut to a size of 5 cm × 5 cm and placed horizontally on the sample table. A droplet of DI water (2.5 μL) was deposited on it and the measurement was completed within 30 s. The reported values were averaged over three measurements.

### 2.2. Configurations

**Figure 1:**
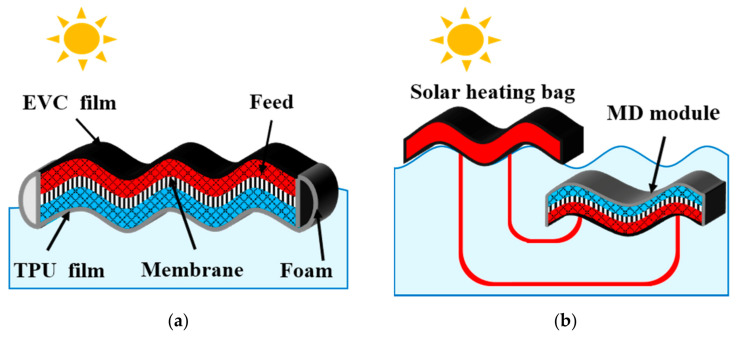
Schematics of the solar-driven MD configurations: (**a**) the simplest configuration; (**b**) the optimal configuration.

Taking AGMD as a prototype, different MD configurations were set up for experiments at the lab scale. Figure 1a shows the simplest configuration. The feed was poured into the MD module through a nozzle and was heated by solar energy directly. The water molecules evaporated from the feed side and moved through the PTFE membrane, and were condensed to liquid water in the air gap. After the experiment, permeate was collected through a nozzle on the permeate side and weighted by a digital mass balance. The MD module was floating on the surface of the coolant water. For this configuration, black EVC film and double-sided transparent tape were used to make a feed chamber filled with polyethylene mesh as support. The area of the EVC film was 100 cm^2^ (10 cm × 10 cm). The thickness of the double-sided transparent tape was 2 mm and the width was 20 mm. The area of the membrane was 100 cm^2^ (10 cm × 10 cm). The effective area of the membrane was ~80 cm^2^. Thermoplastic urethane (TPU) was used as the condensing wall of the MD module. The area of the TPU film was also 100 cm^2^ (10 cm × 10 cm). The double-sided transparent tape was also used in the permeate side to form an air gap which was also filled with polyethylene mesh as support. The air gap thinness (*δ*) was set as 2, 4, 6, and 8 mm respectively. The edge of the MD module was wrapped with PVC foam to make sure that it could float on the coolant water surface. The feed was saline water with 3.5 wt% sodium chloride (NaCl), a salt concentration comparable to the normal seawater.

The optimal configuration with the best performance in terms of permeate flux is shown in Figure 1b. The structure of the MD module was the same as the simplest configuration. The air gap thickness was 4 mm. One of the differences between those two configurations was that, for the optimal configuration, the feed was heated by the solar energy in the solar heating bag instead of the MD module. Another difference was that the MD module of the optimal configuration was completely submerged in the coolant water while the MD module of the simplest configuration was floating on the surface of the coolant water. The surface area of the solar heating bag was 150 cm^2^ (10 cm × 15 cm). The pipes (*d* = 10 mm) were used to connect the solar heating bag with the MD module. The length of the pipes was approxiamtely 10 cm and wrapped with insulation cotton to decrease the waste of energy. The PVC foam was used to keep warm the solar heating bag and the feed side of the MD module. It could also make the solar heating bag float on the coolant water. The NaCl mass concentration was set as 5, 10, 15, and 20 wt% to investigate the performance of the MD module in terms of permeate flux and salt rejection ratio.

To study the performance of the simplest and optimal configurations, a 250 W Philips heating lamp (Incandescent 230–250 V BR125) was used as the solar source. The heating intensity was controlled by adjusting the distance between the lamp and the top surface of the MD module. As in the previous study [29], the frequency of the sea waves was approximately 0.01 to 1 Hz. The experimental device was shaken at a frequency of ~0.03 Hz to simulate the sea waves to investigate the performance of the optimal configuration.

In this work, the membrane was parallel to the coolant water surface, and the direction of evaporation was upward or downward. Another configuration (Figure S3) was tested to find a better performance. The structure of this configuration was also the same as the simplest configuration, but the feed of this module was heated by a water bath, and smoothly circulated by a magnetically driven pump (MP-15R, Guangquan Machinery Co., Ltd., Dalian, China). The feed flow rates (0.4, 0.8, and 1.2 L/min) were controlled by the flow gauge (LZB, Changzhou Shuanghuan Thermo-Technical Instrument Co., Ltd., Changzhou, China). The air gap was set as 2, 4, 6, and 8 mm. The feed was also saline water with 3.5 wt% NaCl as described before. The feed temperature was maintained at 60 °C.

The temperatures of this work were measured by thermometer with thermocouples. The temperature of the coolant water was maintained at 21 ± 1 °C. The salt rejection ratio (*R*) was calculated from the concentrations of chloride ions in the feed side and permeate side.

(1) $$R=1-\frac{{\left[{Cl}^{-}\right]}_{p}}{{\left[{Cl}^{-}\right]}_{f}}$$

where [Cl^−^]*_p_* is the mass concentration of chloride ions in permeate and [Cl^−^]*_f_* is the mass concentration of chloride ions in feed.

### 2.3. Computational Domain and Algorithm

**Figure 2:**
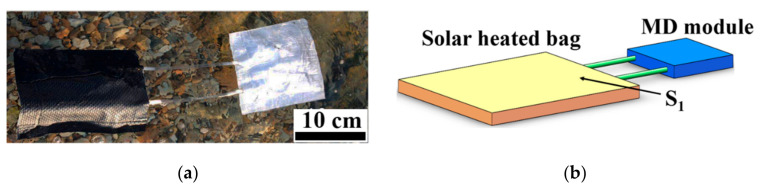
(**a**) The prototype of the experimental unit floating on the water surface; (**b**) the three-dimensional schematic of the test MD unit for numerical simulation.

The computational fluid domain was the feed in the solar heating bag and the MD module. The size of the computational model (Figure 2b) was the same as the size of the experimental device (Figure 2a). Mesh was generated by the commercial software ANSYS^®^ ICEM CFD 18.0, and the structured hexahedral grid was used. The computational fluid was 3.5 wt% NaCl solution. The physical parameters of the salt solution were set according to the previous study [30]. The boundary condition type of the S_1_ surface was velocity-inlet. When there were no waves, *v* = 0 m/s. When there were waves, according to the size of the experimental device and the frequency of the waves, *v* = 0.01 m/s. The boundary condition type of other surfaces, as well as the wall and the heat flux, was set to zero. The simulation was carried out using the software ANSYS^®^ Fluent 18.0, with SIMPLE (Semi-Implicit Method for Pressure Linked Equations) algorithm for pressure–velocity coupling and Second Order Upwind algorithm for the discretization of the conservation equations. The computational accuracy of 10^−3^ was chosen for convergence.

The initial temperature of the fluid (NaCl 3.5 wt%) in the solar heating bag was set as 87 °C. The initial temperature of the fluid (NaCl 3.5 wt%) in the MD module and the connecting pipes was set as 27 °C.

## 3. Results and Discussion

### 3.1. Membrane Characterization

The pore size of the membrane was 0.22 μm. The surface morphology of the membrane is shown in Figure S2a. The LEP of the membrane was ~650 kPa, which meant it was high enough for this work (Figure S2b). The apparent contact angle of the membrane is shown in Figure S2a. The results show that the membrane had high hydrophobicity (149 ± 2°).

### 3.2. MD Configurations and Performance

**Figure 3:**
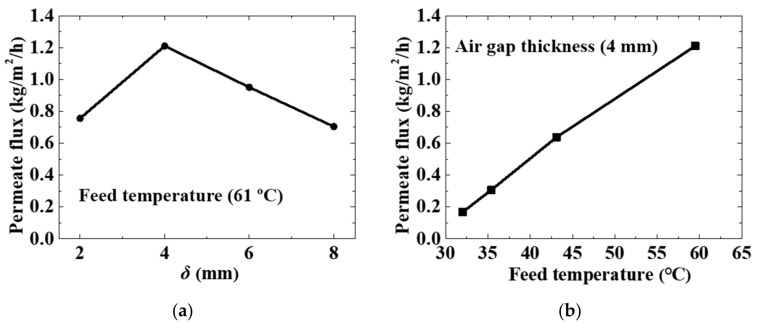
The permeate flux of the simplest configuration under various experimental parameters: (**a**) air gap thickness; (**b**) feed temperature.

The permeate flux of the simplest configuration (Figure 1a) under different experimental parameters is shown in Figure 3. Considering that mass transfer resistance of AGMD mainly comes from the air gap [31], we studied the performance of the simplest configuration when *δ* was 2, 4, 6, and 8 mm, respectively. As shown in Figure 3a, the permeate flux reached its peak value when *δ* was 4 mm. When the air gap thickness was smaller than 4 mm, the permeate flux increased with the increasing air gap thickness. However, according to the previous study [32], the permeate flux decreases with increasing air gap thickness. This discrepancy may have been caused by the structure of the new MD configuration.

Increasing the feed temperature can significantly increase the permeate flux [32]. Our experiment results (Figure 3b) also show that the permeate flux increased dramatically with increasing feed temperature. Figure 3 verifies that the simplest configuration can produce drinkable water only using natural resources (solar energy and seawater). However, the MD module efficiency of the simplest configuration was slightly lower compared to the previous study. It was necessary to enhance the MD module performance.

**Figure 4:**
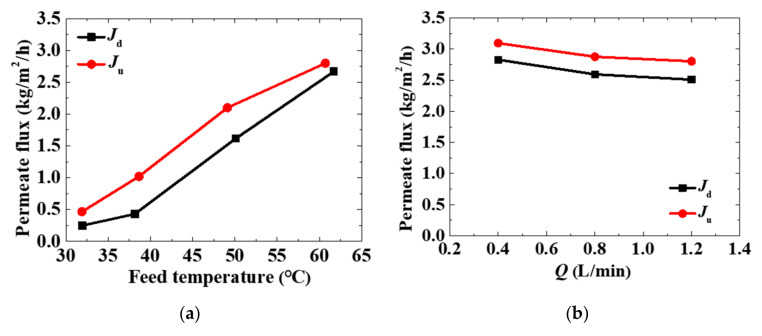
The permeate flux of the verified configuration under various experimental parameters: (**a**) feed temperature; (**b**) feed flow rate. *J*_d_ is the permeate flux when vapor moves downward. *J*_u_ is the permeate flux when vapor moves upward.

As shown in the experiment results of the verified configuration (Figure 4), under the same operation parameters, the permeate flux of upward evaporation (*J*_u_) was always larger than the permeate flux of downward evaporation (*J*_d_), which means upward evaporation is one way to enhance the performance of the simplest configuration.

The feed temperature plays a dominant role in the permeate flux of the MD module. As shown in Figure 4a, the permeate flux increased significantly with increasing feed temperature. Therefore, increasing the feed temperature is another way to enhance the performance of the MD module.

According to the previous study [5], the permeate flux should increase slightly with increasing feed flow rate. Increasing the feed flow rate can enhance the flow turbulence, reduce the thermal polarization near the membrane surface, and enhance the performance in terms of permeate flux of the MD module. However, as shown in Figure 4b, the permeate flux of the MD module decreased slightly with the increasing feed flow rate. This result runs contrary to expectations. It was considered to be caused by the characteristics of the MD configuration. This configuration was completely made of flexible material. The larger the feed flow rate, the larger the possibility of the MD module deformation. When the MD module is deformed, the hydrophobic membrane will be deformed accordingly. The deformation of the membrane reduces its effective area which makes the permeate flux decrease. On a micro-scale, the MD process is a water molecule diffusion behavior through a porous hydrophobic membrane. The mass transfer mechanism in the pores is dominated by the Knudsen number (*K*_n_) [33]:

(2) $$K_{n}=\frac{\lambda }{d}$$

where *λ* is the mean free path of a molecule, and d is the mean pore size of the membrane. The deformation of the membrane changes the shape of the membrane pores and also *d* in Equation (2). After that, the movement path of water vapor molecules in the membrane pores is changed, which reduces the permeate flux. Although the permeate flux decreased slightly with increasing feed flow rate, the smallest value of permeate flux (~2.5 kg/m^2^/h (feed temperature ~60 °C, feed flow rate 1.2 L/min)) under the effect of feed flow rate was still larger than the largest value of permeate flux (~1.2 kg/m^2^/h (feed temperature ~60 °C, feed flow rate ~0 L/min)). It was reasonable to think that using waves to destroy the temperature polarization near the membrane surface was a positive way to enhance the performance of the MD module.

The above study shows that the simplest configuration was available, but the permeate flux was somewhat low. Three methods that can be used to enhance the MD module performance include having the vapor move upward, increasing the feed temperature, and destroying the temperature polarization near the membrane surface. Based on this, we proposed the optimal configuration and investigated its performance under different operating parameters.

### 3.3. Effects of the Sea Waves

**Figure 5:**
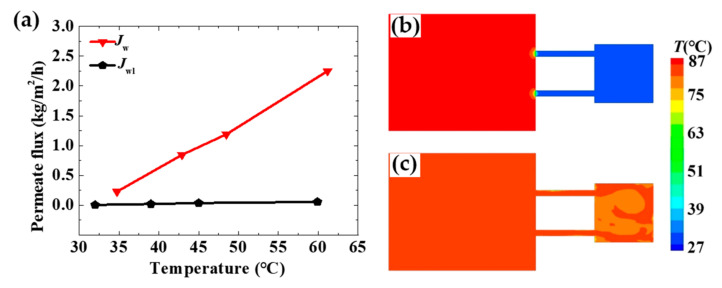
The permeate flux of the experiment and computational temperature with the effect of waves. (**a**) The permeate flux of the optimal configuration in the experiment. *J*_w_ is the permeate flux of the MD module with the effect of the waves. *J*_wl_ is the permeate flux of the MD module without the effect of the waves. (**b**) The computational temperature contour in the cross-section without the effect of waves. (**c**) The computational temperature contour in the cross-section under the effect of waves.

The structure of the MD configuration has a great influence on its performance. For the simplest configuration, both the feed and the permeate are in the MD module. The water vapor molecules moved downward from the feed to the permeate, although its permeate flux was somewhat low (Figure 3). In the verified configuration, the feed is circulated by the pumps between the feed tank and the MD module, which can destroy the temperature polarization and concentration polarization near the surface of the hydrophobic membrane. Hence, the permeate flux of this configuration was larger than the simplest configuration and the optimal configuration. As shown in Figure 5a, *J*_w_ is the permeate flux of the MD module with the effect of the waves and *J*_wl_ is the permeate flux without the effect of the waves in the experiment. The value of *J*_wl_ was almost 0 kg/m^2^/s when there were no waves on the surface of the coolant water. *J*_w_ increased dramatically when the device was shaken at the frequency ~0.03 Hz. The largest value of *J*_w_ was 2.25 kg/m^2^/s. When the device was shaken, the feed could circulate in the solar heating bag and the MD module, which could destroy the thermal boundary layer near the membrane surface. Thus, the performance of the MD could be enhanced.

Figure 5b,c shows the computational temperature contour of the cross-section. The cross-section was parallel to the S1 surface and in the center of the three-dimensional model. When there were no waves, the main method of heat transfer was heat conduction. Because the size of the connecting pipe was much smaller than the size of the solar heating bag, the heat of the fluid was difficult to transfer from the solar heating bag to the MD module. When there were waves, the fluid could cycle in the solar heating bag and the MD module, driven by the momentum. Then, the heat of the fluid was easy to transfer from the solar heating bag to the MD module, the feed temperature was higher, and the permeate flux was larger.

### 3.4. Effect of Salinity

The salt rejection rate (*R*) is another important parameter to evaluate the performance of the MD module. Tables S1–S4 show that *R* in this work was always larger than 99.60% under different operating parameters.

**Figure 6:**
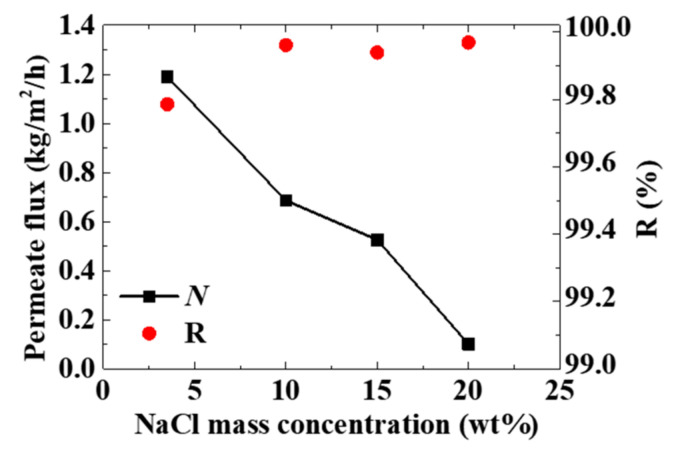
The permeate flux and salt rejection rate under high salt concentrations.

Considering the structural characteristics of the novel MD configuration, as the MD process continues, the salt content of the solar heating bag and the MD module increases. The performance of the optimal configuration under high salt concentration was studied in this work. The feed temperature in the solar heating bag was 50 °C. When the salt concentration of NaCl increased from 3.5 to 20 wt%, the permeate flux decreased dramatically (see Figure 6). The reason for this result is that the larger the salt concentration of the feed, the lower the partial pressure of water vapor in the feed. The vapor pressure difference on both sides of the membrane decreases, which reduces the permeate flux [34]. Although the permeate flux decreased, *R* was still larger than 99.7%. This means the optimal configuration had a good performance under high salt concentrations.

## 4. Conclusions

A novel solar-driven MD device floating on the seawater surface was proposed. It used solar energy to heat the feed, used seawater as the coolant water, and used waves to enhance its performance in terms of permeate flux. The simplest MD configuration was available, whereas the permeate flux of it was somewhat low. The verified MD module shows that upward evaporation lead to a better performance in terms of permeate flux. The largest permeate flux of the optimal MD configuration was 2.25 kg/m^2^/s. The optimal MD module can work under high salt concentrations.

## Supplementary Materials

The following are available online at https://www.mdpi.com/article/10.3390/membranes11050304/s1, Figure S1: Two ways of heating the seawater, Figure S2: Membrane characteristics, Figure S3: Schematics of MD configuration to compare the performance of upward evaporation and downward evaporation, Table S1: Salt rejection ratio of the simplest MD configuration, Table S2: Salt rejection ratio of downward evaporation to verify MD configuration, Table S3: Salt rejection ratio of upward evaporation to verify MD configuration, Table S4: Salt rejection ratio of the optimal MD configuration.
