# Supplementary material for: Experimental Investigation on Floating Solar-Driven Membrane Distillation Desalination Modules
Source: Membranes (Basel). 2021 Apr 21;11(5):304. doi: 10.3390/membranes11050304 (PMC8143288; doi:10.3390/membranes11050304)
Supplement: Supplementary file 1 [file membranes-11-00304-s001.zip › membranes-1180346-supplementary.pdf]

Supplementary materials

Experimental investigation on floating solar-driven membrane distillation desalination modules

Qingxiu Miao, Yaoling Zhang, Shuo Cong, and Fei Guo*

School of Energy and Power Engineering, Dalian University of Technology, Dalian 116024, China
* Correspondence: feiguo@dlut.edu.cn

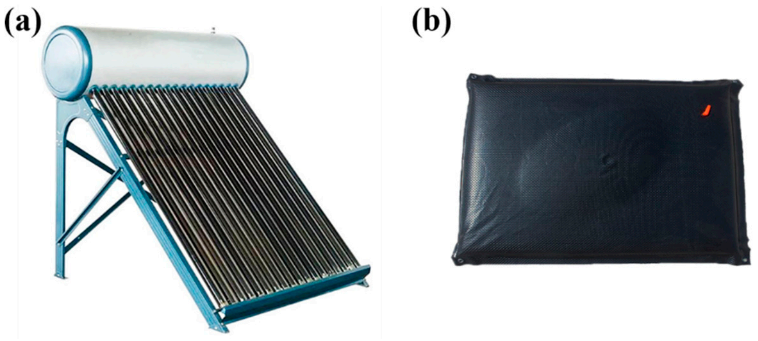

Figure S1. Two ways of heating the sea water. (a) heat pipe solar collector; (b) solar heating bag.

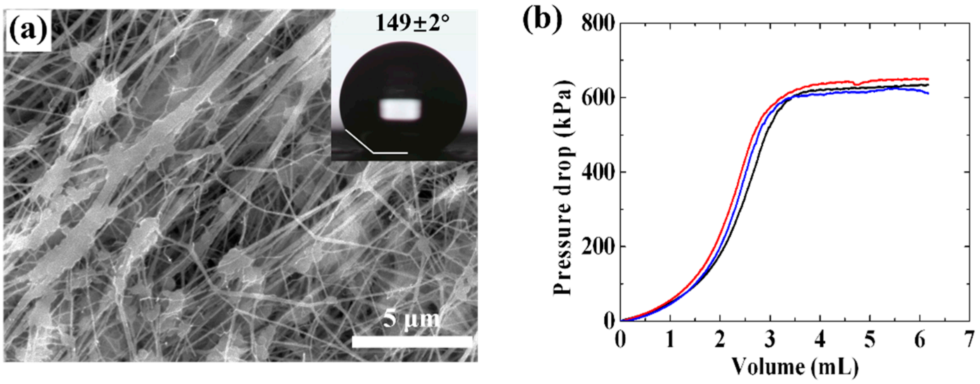

Figure S2. (a) Membrane morphology (pore size: 0.22 μm) and contact angle; (b) LEP measurements of the membranes. The test was repeated three times.

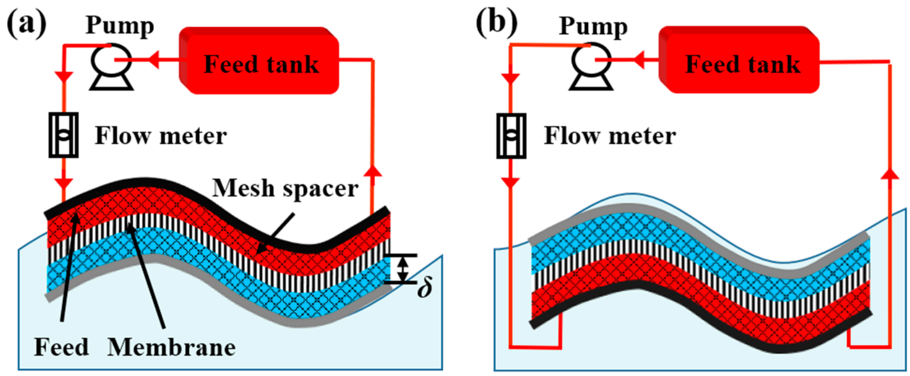

Figure S3. Schematics of varified configuration to compare the performance of upward evaporation and downward evaporation: (a) upward evaporation MD configuration; (b) downward evaporation MD configuration.

Table S1. Salt rejection ratio of the simplest MD configuration.

Feed temperature (ºC)	δ (mm)	R (%)
61	2	99.89
	4	99.79
	6	99.75
	8	99.90
32	4	99.98
35		99.97
43		99.60
60		99.79

Table S2. Salt rejection ratio of downward evaporation verified configuration.

Feed temperature (ºC)	Q (L/min)	R (%)
35	1.2	99.82
43		99.79
48		99.77
61		99.85
60	0.4	99.96
	0.8	99.98
	1.2	99.93

Table S3. Salt rejection ratio of upward evaporation verified configuration.

Feed temperature (ºC)	Q (L/min)	R (%)
32	1.2	99.74
39		99.73
49		99.84
61		99.77
60	0.4	99.89
	0.8	99.88
	1.2	99.78

Table S4. Salt rejection ratio of the optimal MD configuration.

Feed temperature (ºC)	*R* (%)
32	99.99
38	99.90
50	99.79
62	99.78
